# Extraction and generalisation of category-level information during visual statistical learning in autistic people

**DOI:** 10.1371/journal.pone.0286018

**Published:** 2023-06-02

**Authors:** Owen Parsons, Simon Baron-Cohen

**Affiliations:** Autism Research Centre, Department of Psychiatry, University of Cambridge, Cambridge, United Kingdom; Federal University of Paraiba, BRAZIL

## Abstract

**Background:**

We examined whether information extracted during a visual statistical learning task could be generalised from specific exemplars to semantically similar ones. We then looked at whether performance in autistic people differed to non-autistic people during a visual statistical learning task and specifically examined whether differences in performance between groups occurred when sequential information was presented at a semantic level. We did this by assessing recall performance using a two-alternative forced choice paradigm after presenting participants with a sequence of naturalistic scene images.

**Methods:**

125 adult participants (61 participants with an autism diagnosis and 64 non-autistic controls) were presented with a fast serial presentation sequence of images and given a cover task to avoid attention being explicitly drawn to patterns in the underlying sequences. This was followed by a two-alternative forced choice task to assess participants’ implicit recall. Participants were presented with 1 of 3 unique versions of the task, in which the presentation and assessment of statistical regularities was done at either a low feature-based level or a high semantic-based level.

**Results:**

Participants were able to generalise statistical information from specific exemplars to semantically similar ones. There was an overall significant reduction in visual statistical learning in the autistic group but we were unable to determine whether group differences occurred specifically in conditions where the learning of semantic information was required.

**Conclusions:**

These results provide evidence that participants are able to extract statistical information that is presented at the level of specific exemplars and generalise it to semantically similar contexts. We also showed a modest but statistically significant reduction in recall performance in the autistic participants relative to the non-autistic participants.

## Introduction

### Perceptual differences in autism

Autism is diagnosed in approximately 1 to 2% of the population [1–3]. While autism is defined by the presence of social communication difficulties alongside unusually restrictive behaviours, the importance of atypical sensory processing has come to be recognised as a key aspect [4] and is a diagnostic feature of the condition [5].

There is growing evidence of perceptual differences in autistic individuals [6–9]. Attempts to find differences in low-level visual acuity in autistic individuals have failed to provide strong evidence to support this hypothesis [10, 11], suggesting that the widely reported atypicalities in the perceptual experiences of autistic individuals [12] are instead likely be driven by high-level differences in how perceptual information is processed and integrated in the visual system [13].

One account of autism that offers an explanation for these perceptual differences was put forward by Pellicano and Burr [14], who proposed that a Bayesian framework might explain the perceptual atypicalities seen in autism. Their theory, often referred to as the *hypo-priors* account of autism, suggests that autistic individuals may weight their prior expectations to a lesser extent that neurotypical individuals when processing perceptual stimuli, which leads to fewer internal constraints occurring during perceptual inference. Van de Cruys and colleagues [15] argued that there was little evidence to support weakened priors in autism but instead suggested that overfit priors were a more likely candidate mechanism. This variation of a Bayesian account of autistic perception is more closely aligned with previous reports of autistic individuals that reported difficulties in category formation and generalisation [16–19]. If overfitting of priors occurs in autistic individuals, rather than the occurrence of weakened priors, we would expect autistic individuals to perform similarly to non-autistic individuals in tasks that required contextual statistical information to be extracted and applied to similar contexts, but to show reduced relative performance in tasks where acquired statistical information had to be applied to new contexts [15].

### Visual statistical learning of semantic information

A key part of visual perception involves the visual system using previously acquired implicit knowledge of the external world to make inferences about the environment from noisy sensory information [20]. Indeed, because of the often unconscious level at which these perceptual inferences are processed, it has been suggested that one suitable approach to assessing the nature of prior expectations during perception is through statistical learning paradigms [21]. Statistical learning is a domain-general mechanism [22] and has been tested extensively in the visual domain [23–25]. Statistical learning paradigms can be used to create context-based associations and to build expectations of subsequent stimuli, which can increase perceptual attention to surprising (and therefore more informative) stimuli [26]. While visual statistical learning has previously been suggested to be an entirely implicit process [27], it has been shown that some degree of conscious awareness of underlying regularities can occur during visual statistical learning paradigms [28]. Visual statistical learning should instead be viewed as a form of incidental learning rather than purely implicit learning.

Early studies looking into visual statistical learning tended to assess learning using sets of abstract symbols [22–24]. These studies have since been expanded upon to assess whether statistical learning occurs when naturalistic scene images are used instead of symbol shapes [29], focusing on assessing visual statistical learning in a less abstract setting. Brady and Oliva [29] conducted a study that explored whether statistical learning would occur when using these ‘real-world’ stimuli sets. They carried out a number of experiments to assess whether predictive information occurring at higher-levels of abstraction could also be learned in an unsupervised manner. After initially showing that their participants did show robust effects of statistical learning of the transitional associations between real-world scenes, such as pictures of buildings, mountains and forests, they went on to demonstrate that participants were also able to learn predictive information when it occurred primarily at the semantic level. This was done by presenting participants with a sequence of images in which transitions between certain categories of images were more likely, but each individual image was only presented once.

### Visual statistical learning in autistic people

The majority of studies looking into statistical learning in autism have reported an absence of group differences between autistic and non-autistic individuals [30–32]. However, these studies typically focused primarily on motor learning or low-level visual learning. The studies that did focus on visual statistical learning typically used abstract symbols as the stimuli set [30]. The use of abstract symbols without semantic meaning could have led to these studies being insensitive to important variability in how real-world visual information is processed. As autistic individuals have been shown to focus on low-level details to a greater extent [33–36], it is possible that these differences in perceptual processing could affect how higher-level, semantic information is processed. Testing for differences in how the semantic content of higher-level perceptual information is processed could explain the reports of difficulties with generalisation in autism [19] as well as examining the claim that reduced generalisation might occur as a result of a reduced influence of prior information [37].

The present study uses the paradigm developed by Brady and Oliva [29] to assess whether autistic individuals are able to extract statistical information from visual sequences of real-world scenes and whether this effect remains when the statistical regularities are presented at a semantic level. We had three specific questions that we set out to answer. First, we wanted to establish whether participants were able to generalise to semantically similar contexts when statistical information is presented in a fixed context. Understanding whether learning effects were detectable in this paradigm when participants were required to generalise to new contexts would help to inform whether this would be a suitable approach for trying to investigating suggestions of overfit priors in autistic individuals [15]. Second, we wanted to assess whether the reported lack of differences between autistic and non-autistic individuals in visual statistical learning tasks persisted when real-world stimuli were used instead of abstract symbols. Third, we wanted to assess whether autistic and non-autistic participants showed differences in the extent to which they used semantic information when extracting statistical information from their environment. This would help us to understand whether differences in the processing of the semantic content of statistical information in the environment could explain the suggestions that a reduced tendency to generalise in autistic individuals occurs as a result of an attenuated influence of prior information [19, 37].

We tested three specific hypotheses to help us answer these questions using the following approaches:

1. We use an adapted version of Brady and Oliva’s [29] paradigm to test whether we could find statistically significant effects of learning when participants were required to generalise statistical regularities from specific exemplars to semantically similar stimuli.
2. We assessed whether we were able to find evidence of reduced visual statistical learning in autistic individuals when compared to non-autistic individuals specifically when the behavioural task presents participants with real-world stimuli as opposed to abstract visual stimuli.
3. We examined whether the learning effects detected in autistic individuals differed in a statistically significant way from non-autistic individuals when participants were specifically required to process statistical regularities at the semantic level.

## Materials and methods

### Participants

A total of 125 participants took part in this study. This sample comprised of 61 participants with an autism diagnosis (44 males) and 64 non-autistic controls (45 males). Informed consent was obtained from all participants. This study was approved by the Psychology Research Ethics Committee in Cambridge (PREC. 2015.018) and all experiments that were performed met the relevant guidelines and regulations. Participant recruitment and data collection were carried out between December 2015 and December 2018. Identifiable information for study participants was encrypted and stored separately from task data. All participants were right-handed and had normal or corrected-to-normal vision. Participants with a diagnosis of an autism spectrum condition were recruited from the Cambridge Autism Research Database (CARD) and control participants were recruited from the Cambridge Psychology Volunteers Database or through classified adverts on websites such as Gumtree. Before completing the behavioural task, participants were asked to complete the Wechsler Abbreviated Scale Of Intelligence (WASI) as a measure of IQ.

### Stimuli

All stimuli were presented using the Psychtoolbox extension [38, 39] in MATLAB [40]. The task involved presenting participants with various images of real-world scenes. These images were taken from 12 image sets of different scene categories [41]. These stimuli are available from Timothy Brady’s website (http://timbrady.org/stimuli.html). Each set comprised of 68 images of scenes belonging to specific categories. The 12 categories used in this study were: bathrooms, bedrooms, bridges, sky scrapers, coasts, fields, forests, kitchens, living rooms, mountains, roads, and waterfalls. Images were presented in the centre of the screen on a grey background. All images subtended visual angles of 7.5 x 7.5. Participants were seated 60cm from a 24" monitor running at a resolution of 1920x1080.

### Procedure

The task consisted of two distinct phases: a training phase and a recall phase. Participants were not informed that they would have to complete the recall phase until after they had finished the training phase. The instructions given at the start of the task referred only to the training phase and the instructions for the recall phase were only given to participants once the training phase was complete.

#### Training phase

Images of scenes were presented one after another for 300ms with a 700ms interval between images. Unbeknownst to participants, the images from the 12 different categories were randomly arranged into four subsets of three images (triplets). Images within triplets were always presented in a fixed order. The full sequence of images was created by randomly arranging 60 instances of each of the four triplets. The order was constrained so that the same triplet would never appear consecutively and so two triplets would not appear one after another twice in a row (i.e. XYXY would be forbidden, where X and Y represent triplets).

The images that any given participant was exposed to during the training phase were selected in one of two ways; either (i) using *specific exemplars*, where a single image from the stimuli set was assigned for each of the 12 different categories, or (ii) using *generalised exemplars*, where a unique image from the given category was used every time that category was represented in the sequence. When *specific exemplars* were used during training, each triplet structure was represented by a set of 3 specific images that were used every time the triplet was presented. This condition allowed participants to extract information about the triplets by implicitly attending to the specific images. When *generalised exemplars* were used during training, the triplet structure was represented by a novel set of 3 images from the relevant categories each time that triplet was presented. In order for participants to extract information about the triplet structures in this condition, they would be required to implicitly attend to the features or semantic content of the presented image that are consistent across the images in the given category.

All participants completed a total of 770 trials during the training phase. This comprised of 720 standard trials (which consisted of 60 presentations of each of the 4 different triplets) and 50 duplicate trials. Trials were presented across 5 blocks and participants were given a 60-second break between each block.

**Fig 1:**
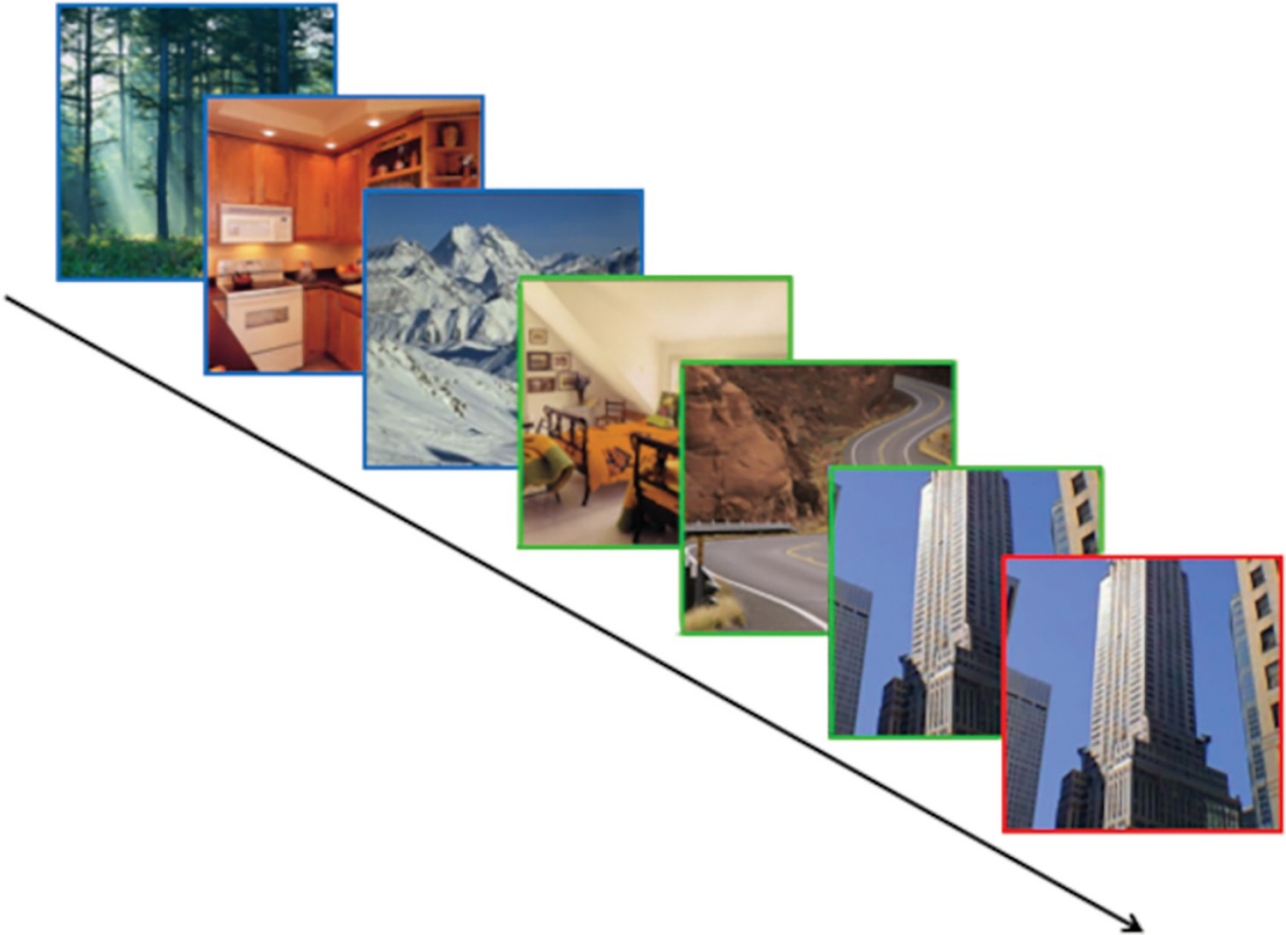
Example sequence. Diagram showing an example sequence of images. Two separate triplets are shown, highlighted in blue and green respectively. A duplicate trial is demonstrated, highlighted in red.

During the training phase, participants were instructed to respond by pressing the space bar when they saw back-to-back repeats of the same image (referred to as a duplicate). It was made clear to participants that they should only respond when they saw the image repeating on the trial that immediately followed the initial presentation. This cover task was utilized to increase attention to stimuli while both reducing the chance of participants becoming explicitly aware of the underlying sequence and providing a measure of attention during the task. To ensure the triplet structure was kept intact, only the first or third images in a triplet could be repeated. This procedure is demonstrated in Fig 1.

#### Recall task

After the training sequence had finished, participants were then informed that they were going to be tested on the familiarity of various three-image sequences. During each test trial, one of the four triplets was presented alongside a foil triplet. Foil triplets were constructed by taking three images from three different triplets. Each of the four triplets was presented alongside each of the four foil triplets twice, with the order of presentation being counterbalanced. This gave a total of 32 trials that were presented in a random order. Each trial consisted of the images within the two triplets (genuine and foil) being presented with the same exposure and inter-stimulus intervals as the training phase. The two sequences (of the genuine and foil triplets) were separated by a 1000ms presentation of a fixation cross. After the genuine and foil triplets had both been presented, participants were then asked to respond to whether they thought the first or second sequence was more familiar by pressing the left or right keys.

Similarly to the training phase, the images participants were presented with during the recall phase were selected either using *specific exemplars* or using *generalised exemplars*. When *specific exemplars* were used in the recall phase, the specific images that were used to represent each category matched the images that were used to represent the categories in the training phase. When *generalised exemplars* were used in the recall phase, a unique image from the given category was used every time that category was represented in the genuine or foil triplets. To identify the genuine triplets when *generalised exemplars* were used during the recall phase, participants were required to have implicitly extracted information about the common features or semantic content that were consistent across the images in a given category. Whereas in the case that *specific exemplars* were used during the recall phase, participants would have only needed to extract information about the specific images used in each triplet to be able to identify the genuine triplets.

### Conditions

**Fig 2:**
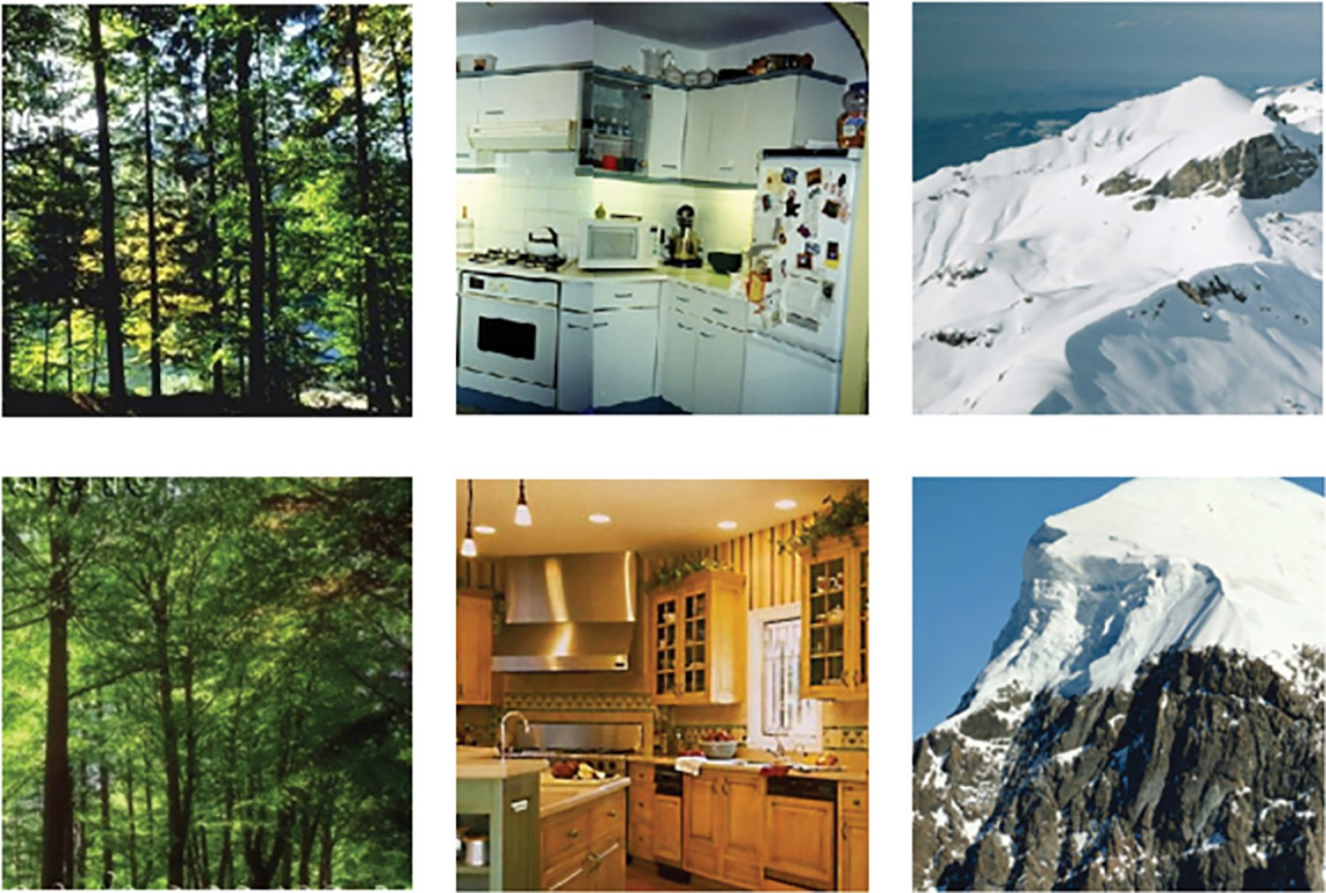
Example stimuli. Two examples of stimuli for the same triplet in the category-level condition. The triplet shown, forest—kitchen—mountain is the same as the blue triplet in Fig 1.

There were three versions of the task to which participants were randomly assigned. These versions of the task were the standard condition, the category condition and the generalisation condition. In the standard condition of the task, *specific exemplars* were used during the training and recall phases. This meant that a single image was taken from each of the 12 different categories and used during both phases of the task. In this condition, participants would see each of these 12 images a number of times and the transitional information (created by the triplet structure) was associated with specific images. For the category condition, *generalised exemplars* were used during the training and recall phases. This meant that a unique image was used each time a category was represented in the sequence. Therefore, each of the presentations of a triplet comprised three novel images but the category these images belonged to stayed constant. Fig 2 shows an example of two instances of the same triplet in the category condition. This meant that participants only saw each individual image once and the transitional information was not associated with specific images but instead with category sets. Finally, the generalisation condition was a hybrid of the other two conditions. In this condition, *specific exemplars* were used during the training phase but *generalised exemplars* were used in the recall phase. This meant that participants were presented with a single image per category during the training phase (as in the standard condition) but were shown novel images during the recall phase (as in the category condition). In this condition, participants would have been presented with the transitional information which was associated with specific images but were then tested on whether they also acquired transitional information associated with category sets. The foil triplets presented in the category condition and generalisation condition were constructed by taking three categories from three different triplets and selecting previously unseen images for the respective categories. While the training procedure differs between the category and generalisation conditions, both conditions require participants to extract higher level information during the training phase in order to perform at an above chance level during the recall phase. Therefore, we can test for more general differences in the ability to extract semantic information between the two diagnosis groups by considering performance across both the category and generalisation conditions.

### Condition assignment

Each participant was assigned to one of the 3 different task conditions. 20 autistic participants (15 male) and 20 non-autistic participants (15 male) completed the standard condition of the task, 20 autistic participants (15 male) and 22 non-autistic participants (16 male) completed the category condition and 21 autistic participants (14 male) and 22 non-autistic participants (14 male) completed the generalisation condition. While the overall study cohort was relatively large (N = 125), the group sample sizes become quite modest when we stratified by both diagnosis group and task condition (ranging between N = 20 and N = 22 participants across the 6 subgroups). A post-hoc power analysis indicated that these sample sizes would only be sufficient to detect large expected effect sizes (Cohen’s d>0.87) with 80% power. However, stratifying by only one of the two factors results in larger subgroups which would allow for 80% power for detecting smaller expected effect sizes (Cohen’s d>0.50 when stratifying by diagnosis group and Cohen’s d>0.63 when stratifying by task condition). To better determine whether null results are indicative of an absence of an effect or are due to lack of statistical power, all null results will be accompanied by corresponding Bayesian tests in order to quantify the relative evidence in support of the null and alternative hypotheses.

### Data analysis

#### Training phase

Initial analysis of performance during the training phase was done using the proportion of correct responses across all trials in the training phase. Responses were coded as correct if there was a response during a duplicate trial or if these was an absence of a response in a non-duplicate trial. Responses were coded as incorrect if there was no response during a duplicate trial or there was a response during a non-duplicate trial. Scores were calculated as the proportion of the 770 trials in which the participant responded correctly.

The overall score is a relatively insensitive measure, as it fails to distinguish between errors that occur due to participants missing duplicates and errors that occur due to participants incorrectly responding during standard trials. To better capture response performance, a Signal Detection Theory [42] approach was used to assess the sensitivity and response criterion of participants responses during the training phase. This was done by treating trials in which duplicate images were presented as trials in which a ‘signal’ was present and all other trials as trials in which only ‘noise’ was present [43]. The sensitivity, or discriminability index (*d*’), is a description of how discriminable the signal is from noise (or the absence of a signal). This can be defined as shown in Eq 1:

$$d^{\prime }=\frac{\mu_{S}-\mu_{N}}{\sqrt{\frac{1}{2}(\sigma_{S}^{2}+\sigma_{N}^{2})}}$$ (Eq 1)

where *μ_S_* and *σ_S_* are the mean and variance for responses in ‘signal trials’ and *μ_N_* and *σ_N_* are the mean and variance for responses in ‘noise trials’. This can also be written as shown in Eq 2:

$$d^{\prime }=Z(hitrate)-Z(falsealarmrate)$$ (Eq 2)

where Z(p) is the inverse of the cumulative distribution function of the given Gaussian distribution. It is also possible to calculate the response criterion, or response bias (*C*), which describes whether participants are biased towards over or under responding during the task. This can be expressed as shown in Eq 3:

$$C=\frac{-Z(hitrate)+Z(falsealarmrate)}{2}$$ (Eq 3)

These two measures were calculated and used for a more detailed assessment of performance during the training phase.

#### Recall phase

Following the methods of Brady and Oliva [29], performance during the recall phase was initially analysed using the proportion of correct responses across all trials. Responses were scored as correct if the participant correctly chose the true triplet and not the foil triplet. Single-sample t-tests were used to gauge whether the responses of participants across the groups as a whole were statistically different from chance guessing. Potential main effects of group and condition, as well as a possible interaction effect between the two variables, were tested for using a two-way ANOVA. The approach used by Brady and Oliva [29] only assessed whether learning effects were present at the level of the group as a whole. As mentioned in the participants section, all null results will be accompanied by corresponding Bayesian tests.

#### Availability of data and materials

Participants were not asked to give consent for their data to be anonymously shared and so the raw data are not available for this study.

## Results

### Comparison of diagnostic groups and participant distributions across task conditions

There were no significant differences between the two diagnostic groups (control and autism) on IQ (Control group, *M* = 118.49, *SD* = 9.95; Autism group, *M* = 115.86, *SD* = 13.16; *t*(123) = 1.27, *p* = .21; *Cohen’s d* = 0.22; *95% CI* [-1.52, 6.78]), age (Control group, *M* = 30.01, *SD* = 7.76; Autism group, *M* = 32.18, *SD* = 8.40; *t*(123) = 1.50, *p* = .13; Cohen’s d = 0.27; *95% CI* [-0.69, 5.03];) or the proportion of males to females (*Χ*^2^(1) = 0.26, *p>*.*30*).

Chi-squared tests were carried out on the frequencies of males and females across the two diagnostic groups for each of the 3 task conditions. These were found to be non-significant for the standard (*Χ*^2^(1) = 0.0, *p>*.*30*), category (*Χ*^2^(1) = 0.03, *p>*.*30*) and generalisation conditions (*Χ*^2^(1) = 0.01, *p>*.*30*), suggesting the ratio of male and female participants was balanced across the autism and control groups for all conditions. Similarly, there were no differences in the overall proportion of males and females (*Χ*^2^(2) = 1.20, *p>*.*30*) or the proportion of autistic to non-autistic participants (*Χ*^2^(2) = 0.05, *p>*.*30*) across the 3 conditions.

Participants in the 3 different conditions were also assessed for differences in age and IQ. When considering age, there were no differences between the autistic and non-autistic participants in the standard condition (*t*(38) = 0.92, *p>*.*30*; *Cohen’s d* = 0.31; *95% CI* [-1.96, 6.04]), the category condition (*t*(40) = 0.67, *p>*.*30*; *Cohen’s d* = 0.27; *95% CI* [-2.53, 6.63]) or the generalisation condition (*t*(41) = 1.10, *p =* .28; *Cohen’s d* = 0.34; *95% CI* [-1.71, 5.65]). Similarly for IQ, no differences were found between autistic and non-autistic participants in the standard condition (*t*(38) = 0.43, *p>*.*30; Cohen’s d = 0*.*39; 95% CI [-2*.*65*, *10*.*95])*, category condition (*t*(40) = 0.19, *p>*.*30; Cohen’s d = 0*.*06; 95% CI [-6*.*76*, *8*.*32]*) or generalisation condition (*t*(41) = 0.28, *p>*.*30; Cohen’s d = 0*.*40; 95% CI [-2*.*76*, *13*.*16])*. There were also no overall differences between the three conditions on either age (*F*(2,122) = 1.05, *p>*.*30; η*^*2*^ *= 0*.*02*) or IQ (*F*(2,122) = 1.06, *p>*.*30; η*^*2*^ *= 0*.*01*).

### Training phase

**Fig 3:**
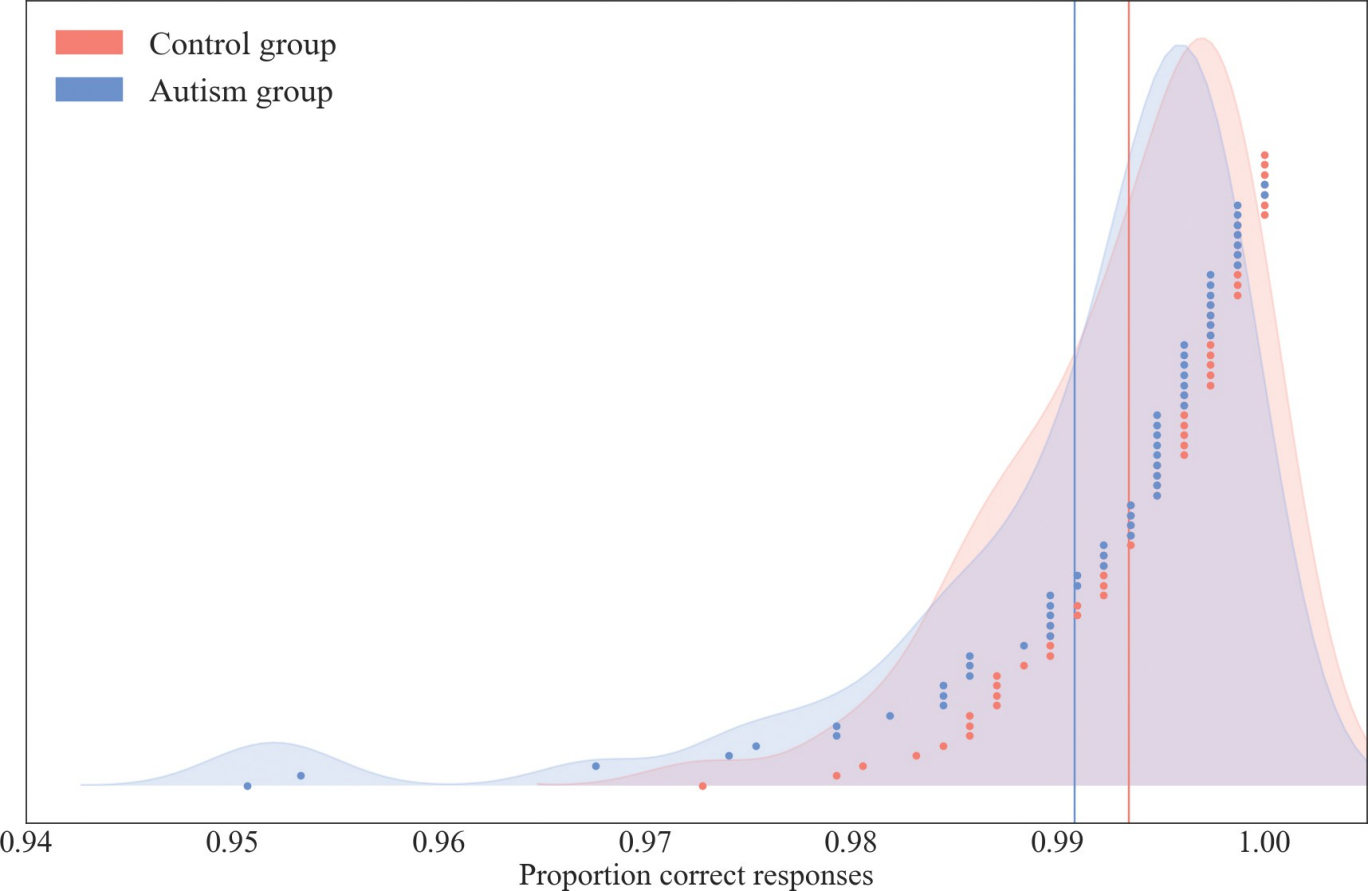
Distributions of scores for training phase. Distributions of scores (proportion correct responses) in the training phase of the task. Distributions are shown separately for the two diagnosis groups and individual data points are shown on top in ascending order from left to right. Group means are shown by the two vertical lines.

Performance during the training phase was initially analysed using the proportion of correct responses across all trials in the training phase. The two groups were found to have equal variances (*F* = 1.86, *p =* .17*; η*^*2*^ *= 0*.*03*) and a t-test indicated that the control participants (*M* = 0.99, *SD* = 0.01) and autistic participants (*M* = 0.99, *SD* = 0.01) did not significantly differ (*t*(123) = 1.82, *p =* .07; *Cohen’s d = 0*.*35; 95% CI [0*.*00*, *0*.*01]*). Group distributions and individual participant scores are displayed in Fig 3.

**Fig 4:**
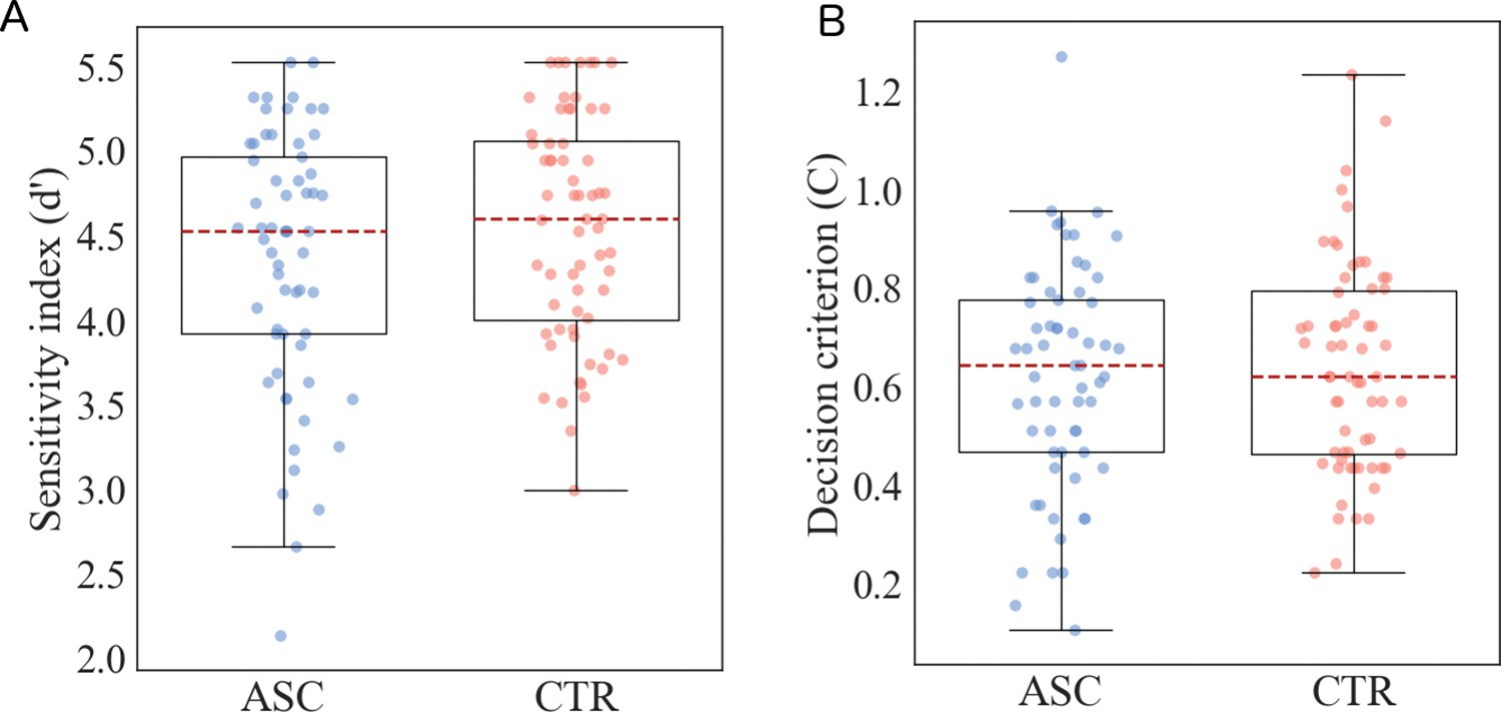
Distributions of sensitivity index and decision criterion. Box plots showing the signal detection performance measures during the training phase. Box plots are shown separately for the two diagnosis groups (control: CTR and autism: ASC), for both the sensitivity index (a) and the decision criterion (b). Individual data from all participants are also displayed.

A signal detection approach was then used to calculate the sensitivity and potential bias in participants responses (as detailed in the methods section). Scores for the sensitivity index (*d*’) and decision criterion (*C*) were calculated for all participants and were compared across the two groups using t-tests. The two groups were found to have equal variances for both sensitivity index scores (*F* = 0.44, *p>*.*30; η*^*2*^ *= 0*.*02*) and decision criterion scores (*F* = 0.18, *p>*.*30; η*^*2*^ *= 0*.*00*). For the sensitivity index scores, the t-test found that the control participants (*M* = 4.56, *SD* = 0.66) and autistic participants (*M* = 4.36, *SD* = 0.77) did not significantly differ (*t*(123) = 1.56, *p =* .12; *Cohen’s d = 0*.*04; 95% CI [-0*.*39*, *0*.*31]*). Similarly, for decision criterion scores the t-test showed that the control participants (*M* = 0.64, *SD* = 0.21) and autistic participants (*M* = 0.62, *SD* = 0.23) did not differ significantly (*t*(123) = 0.45, *p>*.*30; Cohen’s d = 0*.*30; 95% CI [-0*.*66*, *0*.*05]*). These scores are displayed for all participants in Fig 4.

Finally, an additional analysis was carried out to check there were no differences in performance during the standard training (where a single image was presented for each category type) and the category-level training (where a set of images was presented for each category type). This was done by assessing both the sensitivity index and decision criterion scores independently for each of the types of training. No significant differences were found between performance in the standard and category-level training tasks for the control participants scores on the sensitivity index (*t*(123) = 0.59, *p>*.*30; Cohen’s d = 0*.*10; 95% CI [-0*.*50*, *0*.*71])* or decision criterion (*t*(123) = 0.47, *p>*.*30; Cohen’s d = 0*.*19; 95% CI [-0*.*41*, *0*.*81]*). Similarly, no significant differences were found between performance in the standard and category-level training tasks for the autistic participants scores on the sensitivity index (*t*(123) = 1.31, *p =* .*20; Cohen’s d = 0*.*47; 95% CI [-1*.*10*, *0*.*16])* or decision criterion (*t*(123) = 0.60, *p>*.*30; Cohen’s d = 0*.*18; 95% CI [-0*.*44*, *0*.*80]*).

### Recall phase

Performance during the recall phase was initially analysed using the proportion of correct responses across all trials. To assess whether performance during the recall phase was associated with the participants previous performance in the training phase, the correlations between the scores obtained in the two phases were calculated across all participants in each of the two groups.

**Fig 5:**
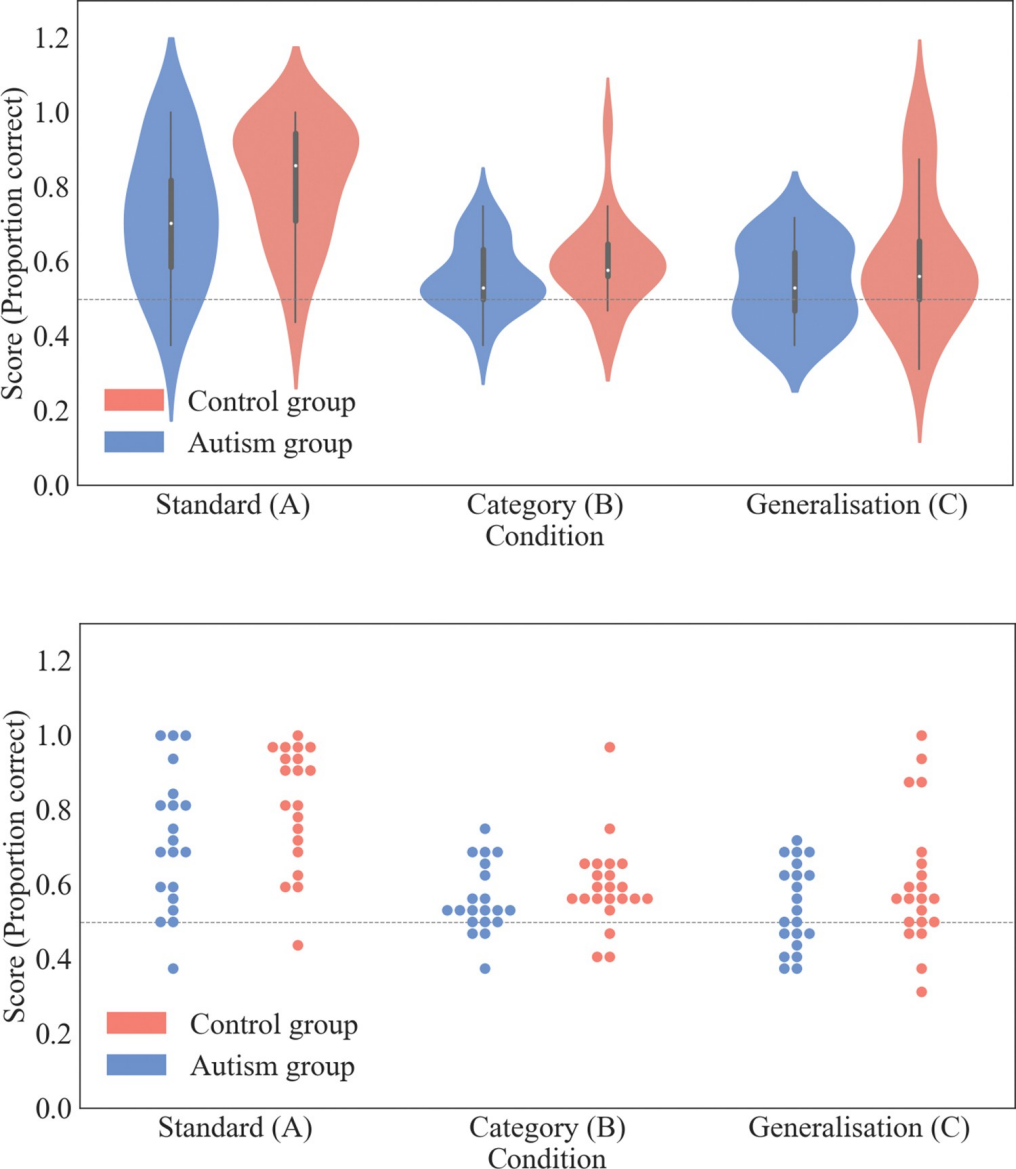
Distributions of scores from the recall phase. Scores in the recall phase (proportion correct) stratified by diagnosis group and condition type. Distributions are shown as violin plots (top) and as individual data points (bottom). The expected score based on chance guessing (0.5) is shown as a horizontal grey dashed line.

To assess whether there were differences in the extent to which the participants showed knowledge of the underlying statistics of the triplets, scores on during the recall phases was compared between participants in the two groups and across the 3 different conditions. Scores obtained by participants in both groups are shown across the different conditions in Fig 5. The main effects of group and condition, as well as a potential interaction effect between the two variables, was tested for using analysis of variance. However, single-sample t-tests were carried out prior to conducting the main analysis to replicate the approach used by Brady and Oliva [29]. This was in order to gauge whether the responses of participants across the groups as a whole were statistically different from chance guessing.

#### Correlation with training phase

**Fig 6:**
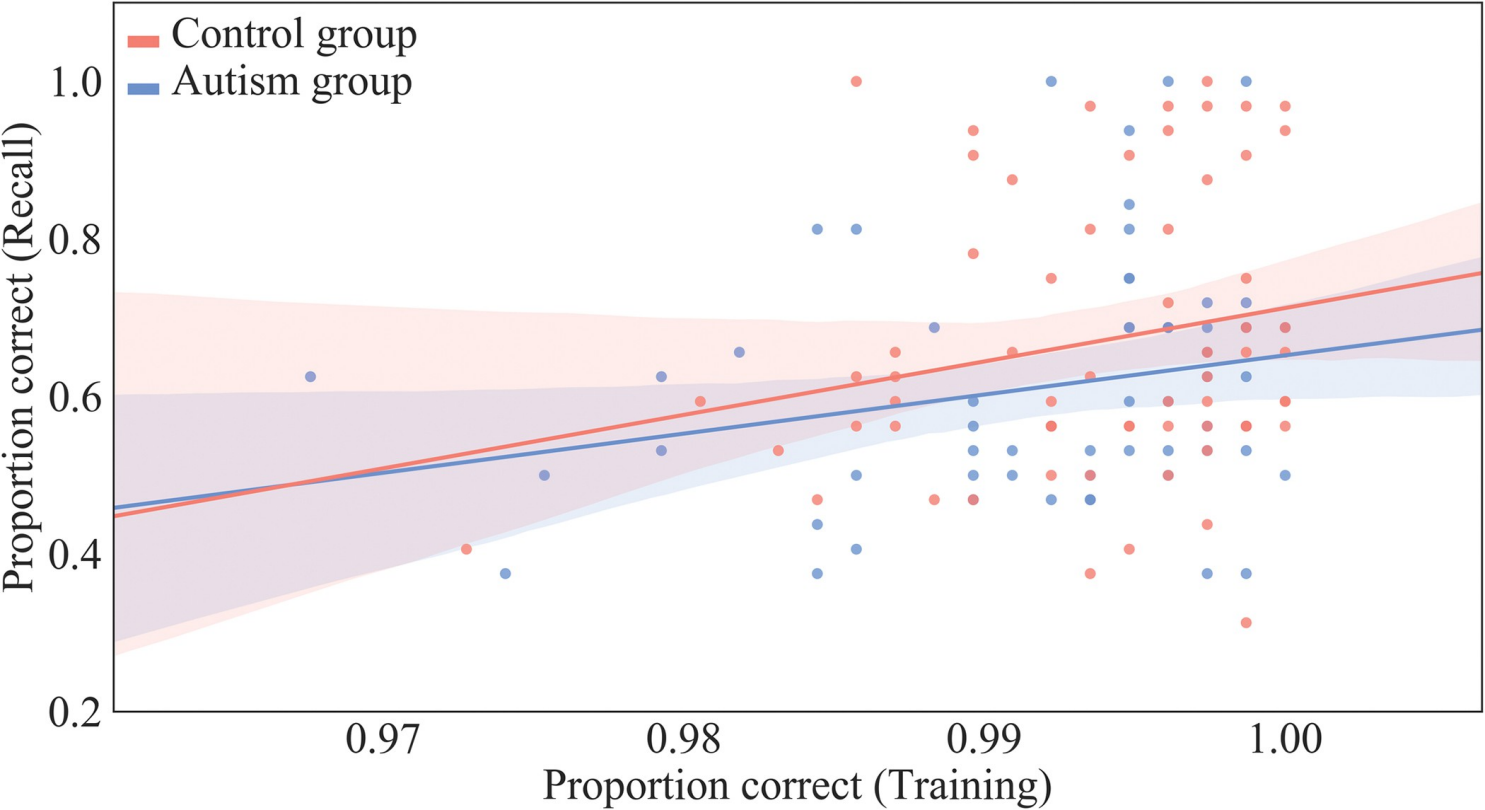
Correlation between training phase and recall phase performance. Scatter plot showing the correlations between performance during the training phase and recall phase for both diagnosis groups. Lines of best fit are shown for each group with the 95% confidence interval displayed by the shaded regions.

Two participants in the autism group had scores in the training phase that were more than 3 standard deviations below the mean and were removed from the present analysis. The correlation between the two performance measures was found to be non-significant within both the control group (*r =* .21, *p =* .*10*) and the autism group (*r =* .22, *p =* .09). A scatter plot showing the associations between performance in the training and recall phases is displayed in Fig 6.

To assess whether this relationship differed across the different condition types, correlations between performance in the training and recall phases were also examined after stratifying the data based on condition. There was a significant correlation between training and recall performance in the standard condition (*r =* .46, *p =* .003) and the category condition (*r =* .41, *p =* .006). While the correlation in the generalisation condition failed to reach significance (*r =* .14, *p>*.*30*), Fisher transforms were carried out and found that the relationship between training and recall performance did not differ significantly between the generalisation condition and either of the other two conditions (Standard condition: *Z* = 1.59, *p =* .11, *two-tailed test*; Category condition: *Z* = 1.34, *p =* .18, *two-tailed test*).

#### Evidence for learning effects

The generalisation condition was considered first as it was not included in the study by Brady and Oliva [29] and was therefore a novel condition. We initially tested for a learning effect across all participants that completed the generalisation condition, including both participants with and without autism in the analysis. The performance of all participants that completed the generalisation condition of the task supported the case that there was a true learning effect (*t*(41) = 3.22, *p =* .002; *Cohen’s d = 0*.*50; 95% CI [0*.*17*, *0*.*81]*). Similarly, learning effects were found for the standard condition (*t*(39) = 9.57,*p*<0.001; *Cohen’s d = 1*.*51; 95% CI [1*.*05*, *1*.*97]*) and the category condition (*t*(41) = 4.88, *p*<0.001; *Cohen’s d = 0*.*75; 95% CI [0*.*41*, *1*.*09]*) as were previously reported by Brady and Oliva [29]. These effects were explored further by conducting additional one-sample t-tests for each of the two diagnosis groups separately. In the standard condition, there was a significant learning effect for both the control participants (*t*(19) = 8.74, *p*<0.001; *Cohen’s d = 1*.*95; 95% CI [1*.*19*, *2*.*70]*) and the autistic participants (*t*(19) = 5.39, *p*<0.001; *Cohen’s d = 1*.*20; 95% CI [0*.*61*, *1*.*77]*). Similarly, for the category condition there were significant effects in both control group (*t*(21) = 3.92, *p =* .008; *Cohen’s d = 0*.*84; 95% CI [0*.*34*, *1*.*32]*) and autism group (*t*(19) = 2.92, *p =* .009; *Cohen’s d = 0*.*65; 95% CI [0*.*16*, *1*.*13]*). In the generalisation condition, there was a significant effect for the control group (*t*(20) = 2.74, *p =* .013; *Cohen’s d = 0*.*60; 95% CI [0*.*13*, *1*.*06]*) but not the autism group (*t*(20) = 1.75, *p =* .09; *Cohen’s d = 0*.*38; 95% CI [-0*.*07*, *0*.*82]*). As we failed to find a significant learning effect in the sample of autistic participants that completed the generalisation condition, we followed up by carrying out a Bayesian equivalent of a one-sample t-test to evaluate whether there was evidence to support the null hypothesis that performance was at chance level. The Bayes factor from the test indicated that there was very weak evidence in favour of a learning effect (*BF*_*10* =_ 1.57), meaning there is a lack of evidence to suggest acceptance of the null hypothesis. It is important to note that these one-sample t-tests were conducted to match the approach taken by Brady and Oliva [29] and Bonferroni corrections were not applied.

#### Effects of diagnosis group and task condition on performance

To test for whether there were differences in performance across the different conditions and between the two groups, an analysis of variance was conducted. A two-way ANOVA was carried out with the proportion of correct responses during the recall phase as the dependent variable and both task condition and diagnostic group as between-subjects independent variables. The results of the ANOVA found significant main effects of condition (*F*(1,118) = 23.43, *p*<0.001*; η*^*2*^ *= 0*.*27*) and group (*F*(1,118) = 6.15, *p =* .015*; η*^*2*^ *= 0*.*04*). Overall, control participants (*M* = 0.67, *SD* = 0.18) tended to identify the correct triplet in a higher proportion of trials than participants in autism group (*M* = 0.61, *SD* = 0.16). The interaction effect between condition and group was non-significant (*F*(1,118) = 0.41, *p>*.*30; η*^*2*^ *= 0*.*00*). Again, as we found a non-significant result we followed this up with a Bayesian equivalent to determine whether there was evidence in support of the null hypothesis that there is no interaction between diagnosis group and task condition. While the result of this additional analysis suggested evidence in favour of the null (BF_01_ = 1.96) the value of the Bayes factor indicates that there is unsubstantial evidence in support of the null hypothesis [44]. The full output table from the ANOVA is shown in S1 Table of the supplementary materials.

A post-hoc comparison was carried out using pairwise t-tests (Bonferroni corrected) to assess the main effect of condition. Scores in the standard condition (*M* = 0.77, *SD* = 0.18) were significantly higher than scores in both the category condition (*M* = 0.58, *SD* = 0.11; *t*(80) = 5.89, *p*<0.001, *Cohen’s d* = 1.30) and scores in the generalisation condition (*M* = 0.575, *SD* = 0.151; *t*(80) = 6.01, *p*<0.001, *Cohen’s d* = 1.17). Scores in the category and generalisation conditions did not significantly differ from one another (*t*(80) = 0.11, *p>*.*30*, *Cohen’s d* = 0.03). This non-significant result was followed up with a Bayesian independent samples t-test between the category and generalisation conditions. The result indicated modest evidence in support of the null hypothesis that the population means are the same between the two task conditions (*BF*_*01*_ = 4.35). The full table for the post-hoc analysis can be found in the S2 Table of the supplementary material.

#### Potential confound of sex as a factor

In addition to ensuring that the distribution of males and females was balanced across diagnostic groups and task conditions, we carried out tests for effects of sex on all the main outcome variables considered in the study. This was done to further validate that sex was not a potential confound in the study. We found non-significant effects for proportion of correct answers during the training phase (*F*(1,122) = 0.06, *p>*.*30; η*^*2*^ *= 0*.*00*), sensitivity index (d’) during the training phase (*F*(1,122) = 0.56, *p>*.*30; η*^*2*^ *= 0*.*01*), decision criterion (C) during the training phase (*F*(1,122) = 0.19, *p>*.*30; η*^*2*^ *= 0*.*00*) and the proportion of correct responses during the recall phase (*F*(1,122) = 0.15, *p>*.*30; η*^*2*^ *= 0*.*00*). Full results from these ANOVA tests are detailed in S3 Table of the supplementary materials.

## Discussion

The paradigm used in this study was designed to assess 3 key hypotheses about how autistic and non-autistic individuals are able to extract associative information at higher-levels of perception. First, we used a novel, adapted version of Brady and Oliva’s [29] task to assess whether semantic information was extracted from stimuli even when the statistical regularities occurred at the level of the individual exemplar. We found evidence for effects of learning in this condition, which indicated that participants were able to generalise learning from a specific context to a broader context. Second, we compared the overall performance of all autistic and non-autistic participants in our sample and found that the autistic participants had statistically significant worse recall than non-autistic participants. Finally, we examined whether autistic participants extracted semantic information from real-world stimuli to a lesser degree than non-autistic participants but were unable to draw any conclusions from out data regarding this hypothesis as we found insufficient evidence either for or against this claim.

Our generalisation condition was a novel version of this paradigm that extended on the work of Brady and Oliva [29]. We found that participants showed above chance recall in identifying sequences of novel stimuli that shared semantic information with the fixed set of exemplars that were presented to them during the training phase. This result indicates that semantic information can be extracted during a visual statistical learning paradigm even if the sequential information occurs at the level of the individual exemplar during the training procedure. Despite the fact that the statistical information contained in the training sequences was not at the semantic level, participants still extracted this semantic information and were able to generalise from a specific context to a general context during the recall phase. Interestingly, our Bayesian analysis found moderate evidence in support of a lack of group differences between our category condition and generalisation condition, indicating that the difference in the training procedure between these two conditions had little effect on performance in the recall phase.

As a significant overall difference in recall was found between our groups, when testing across all 3 task conditions, we can consider how this finding fits in with the wider literature. While a lack of group differences between autistic and neurotypical controls is common across the statistical learning literature, some studies have reported reduced performance in autistic individuals. Such results could be the result of other deficits in autism and not statistical learning ability *per se*. For example, motor abilities can be impaired in autism [45], which might explain reports of impaired learning in motor-sequence tasks [46].

Foti and colleagues [47] carried out a meta-analysis of studies that examined incidental learning in autism and found no differences when combining the results of 11 different studies. This was followed up by an additional meta-analysis [48] which set out to build on and overcome the methodological issues of the study by Foti and colleagues [47]. The concerns that were raised regarding the results by Foti and colleagues [47] were due to the fact that their measures of statistical learning were based on the extent to which reaction times reduced across blocks in which participants were presented with predictive sequences, rather than the more standard approach of comparing differences between blocks where predictive sequences occur and blocks where trials are random [49]. However, the follow up study still did not find any differences between the autistic and non-autistic participants after correcting these methods [48].

A study that looked at visuospatial statistical learning in autism reported superior task performance in autistic adults but not in autistic children [50], but it should be noted that the sample sizes used were very modest. Jones and colleagues [51] looked at visual statistical learning in a large sample of autistic children vs typical controls. When they compared performance across participants in the two groups, no differences were found. However, when they used a discriminant function to test the similarity of the autistic participants’ responses to the typically developing children’s responses, they found two distinct subgroups within the autism group. They also found that the autistic subgroup that performed similarly to the typically developing children tended to have reduced levels of autistic symptoms compared to the other autism subgroup.

The present study includes a large sample of participants and found evidence to suggest that autistic individuals do show reduced learning during our visual statistical learning task. If the results are taken as an example of reduced statistical learning in autistic individuals, it is important to note the specific nature of the task presented in the present study. The type of stimuli used in the tasks presented here should be highlighted, as the majority of previous studies looking at visual statistical learning in autism have used simple, abstract shapes. Brady and Oliva [29] found a series of results that suggest statistical learning can occur in typical individuals for scene-based stimuli, however this is the first study that we are aware of to use such stimuli in a group of autistic participants. The recognition and categorisation of scenes is thought to involve the processing of feature-based statistics which help us to infer the type of scene we are viewing [52].

There is a body of evidence that shows that typically developed individuals are able to process and categorise the overall semantic content of scenes quickly but show limitations when detailed feature representation is required [53]. Based on reports of a tendency of autistic individuals to focus on low-level details to a greater extent [33–36], it is possible that these differences would affect more complex stimuli are processed during rapid serial presentation tasks such as the one presented in this study. While differences in scene processing have been reported in autistic individuals, the stimuli used in these studies tends to include social features such as faces and therefore it is unclear whether such differences are specific to social scenes or extend to non-social scenes as well [54]. A better understanding of how non-social scenes are processed by autistic individuals, and whether any atypicalities do exist, would enable us to determine whether the results of the present study are indicative of a more general deficit in visual statistical learning or whether the result is specific to the stimuli used. Our study was unable to provide meaningful evidence to support or refute the claim that visual statistical learning of semantic information occurs to a lesser degree in autistic individuals. Future research could focus specifically on this hypothesis to allow for larger cohorts to be recruited but it may be the case that any effect found would be too small to be meaningful.

We recognise that there were some assumptions and limitations to the study which we like to address here so that they can be considered in future research. It is important to note that the interpretation of these results rests on the assumption that the approach used in the recall phase accurately captures the level of learning that occurred in the training phase. A 2-alternative forced-choice (2AFC) paradigm was used to assess participants memory for triplets in the recall phase [55]. There are some criticisms of the 2AFC approach that suggest order effects can influence participants’ responses, but these issues are more problematic when testing for perceptual sensitivities rather than recall [56]. An alternative method for assessing recall is the yes/no paradigm [57], but this approach could potentially be problematic when comparing autistic and non-autistic individuals due to differences in the interpretation of the task which could result in biases [58]. Broadly speaking, there are a number of issues that can occur with explicit test phases at the end of statistical learning tasks, such as large amounts of noise when averaging across participants due to chance guessing and less power to detect true effects due to insufficient numbers of trials [59]. Nonetheless, the present finding seems to suggest that autistic individuals extract predictive information from their environment to a slightly lesser extent than non-autistic individuals.

It is also important not to assume that the learning effects found in the category and generalisation conditions were based on semantic information, as it is possible that the higher levels of correlation between low-level features within same-category images compared to different-category images led to the observed recall effects [52]. While, Brady and Oliva [29] carried out experiments specifically to show that semantic information was processed during the task, this does not necessary extend to the sample in the present study. Indeed, it is a possibility that the two groups showed similar recall effects while actually processing different levels of information within the task. This possibility could be clarified by the inclusion of a word-based recall phase, as was included in the original study by Brady and Oliva [29]. However, the present study was unable to include this due to the additional demand on data collection that the inclusion of further conditions would have resulted in. An alternative approach would be to assess whether within-category correlations of low-level features accounted for variation in the observed memorability of the different categories and, if so, whether this effect differed between the two groups [60–62]. This could be explored in future studies to build a more complete picture of the level at which autistic and non-autistic individuals process category-level information.

While the overall sample size of the full cohort was fairly large for a behavioural study, the present study was limited by the fact that the sample sizes reduced when we stratified the full sample into smaller subsamples based both on task condition and diagnostic status. Recruitment was conducted such that the full sample of participants comprised 6 different subsamples of participants, each of which contained only autistic or control participants who completed one of each of the 3 different task conditions. Significant effects of learning were found across the different subsamples of participants, except for the autistic participants who completed the generalisation condition. We computed a Bayes factor for this condition to evaluate whether the data provide evidence for a lack of learning effect but this determined the evidenced was unsubstantial. Similarly, statistical power was a concern when testing for interaction effects between diagnostic group and task condition due to the sample sizes for these stratified subgroups being fairly modest. Again, non-significant results were followed up with Bayesian analysis to determine whether there was evidence in support of the null or whether the evidence was unsubstantial. In cases where we had unsubstantial evidence, we were unable to draw any conclusions about these particular hypotheses. This meant we were unable draw any conclusions regarding whether autistic participants performed differently to non-autistic participants specifically when semantic information was required to be extracted from the stimuli.

Finally, it is important to highlight that high level perceptual differences in how sensory information is processed in autistic individuals should be viewed as differences in the style of perceptual processing rather than deficits. Instead of regarding autistic individuals as ‘better’ or ‘worse’ at aspects of perception, it can be helpful to think of them as having different processing styles that are beneficial in certain situations but detrimental in others. Indeed, suggestions of increased discrimination of similar stimuli [63, 64] that are potentially associated with difficulties in category formation and generalisation [16–19] are also linked to the commonly reported occurrences of heightened perceptual sensitivity that lead to superior performance in autistic individuals compared to non-autistic individuals [7–9]. An important question to answer, should future studies find further evidence to suggest that autistic individuals show a reduction in the extent to which they extract statistical regularities from the environment, is whether the reduced reliance on expected properties of visual stimuli is directly linked to an increase in perceptual sensitivity.

## Conclusions

We found group differences in the ability to recall incidentally learnt sequences of scene images, with autistic individuals showing marginally, but statistically significantly, worse performance during the recall phase of a visual statistical learning task. This is in line with accounts that suggest autistic individuals use prior information to a lesser extent during visual perception. It also supports the view that autistic people tend to generalise less when data might be deemed to be insufficient. This tendency can be viewed as a cognitive style that favours specificity and detail over generalisation, which might be advantageous under certain conditions. We were unable to draw any conclusions from our data regarding group differences when participants were required to extract semantic associations and so were unable to provide evidence either against or in support of the suggestion that autistic individuals overfit priors or the suggestion that they struggle with category formation and generalisation.

## Supporting information

S1 Checklist STROBE statement—checklist of items that should be included in reports of observational studies. (PDF)

S1 Table Full results from the 2-way ANOVA with the proportion of correct responses as the dependent variable and both ‘Group’ (autism or control) and ‘Condition’ (standard, category or generalisation) as between-subject measures. (DOCX)

S2 Table Analysis from the categorical statistical learning task. Post Hoc comparisons of performance across condition types in the recall phase.
(DOCX)

S3 Table Results for tests for effects of sex across all dependent variables examined in the main manuscript. (DOCX)
