# Supplementary material for: Extraction and generalisation of category-level information during visual statistical learning in autistic people
Source: PLoS One. 2023 Jun 2;18(6):e0286018. doi: 10.1371/journal.pone.0286018 (PMC10237412; doi:10.1371/journal.pone.0286018)
Supplement: S1 Table — (DOCX) [file pone.0286018.s002.docx]

Supplementary Table 1: Analysis from the categorical statistical learning task. Full results from the 2-way ANOVA with the proportion of correct responses as the dependent variable and both ‘Group’ (autism or control) and ‘Condition’ (standard, category or generalisation) as between-subject measures.

| Cases | Sum of Squares | df | Mean Square | F | p |
| --- | --- | --- | --- | --- | --- |
| Group | 0.129 | 1 | 0.129 | 6.148 | 0.015 |
| Condition | 0.981 | 2 | 0.490 | 23.426 | < .001 |
| Group * Condition | 0.017 | 2 | 0.009 | 0.413 | 0.663 |
| Residual | 2.469 | 118 | 0.021 |  |  |
