# Supplementary material for: Extraction and generalisation of category-level information during visual statistical learning in autistic people
Source: PLoS One. 2023 Jun 2;18(6):e0286018. doi: 10.1371/journal.pone.0286018 (PMC10237412; doi:10.1371/journal.pone.0286018)
Supplement: S2 Table — Post Hoc comparisons of performance across condition types in the recall phase. (DOCX) [file pone.0286018.s003.docx]

Supplementary Table 2: Analysis from the categorical statistical learning task. Post Hoc comparisons of performance across condition types in the recall phase.

|  |  | Mean Difference | SE | t | Cohen’s d | p |
| --- | --- | --- | --- | --- | --- | --- |
| Standard | Category | 0.188 | 0.032 | 5.892 | 1.303 | < .001 |
|  | Generalisation | 0.192 | 0.032 | 6.009 | 1.170 | < .001 |
| Category | Generalisation | 0.004 | 0.032 | 0.114 | 0.028 | 1.000 |
