# Supplementary material for: Extraction and generalisation of category-level information during visual statistical learning in autistic people
Source: PLoS One. 2023 Jun 2;18(6):e0286018. doi: 10.1371/journal.pone.0286018 (PMC10237412; doi:10.1371/journal.pone.0286018)
Supplement: S3 Table — (DOCX) [file pone.0286018.s004.docx]

Supplementary Table 3: Results for tests for effects of sex across all dependent variables examined in the main manuscript.

| DV | Statistic |
| --- | --- |
| Proportion of correct responses during the training phase | *F*(1,122)=0.055, *p=*0.815 |
| Sensitivity index (d’) during the training phase | *F*(1,122)=0.565, *p=*0.454 |
| Decision criterion (C) during the training phase | *F*(1,122)=0.189, *p=*0.664 |
| Proportion of correct responses during the recall phase | *F*(1,122)=0.148, *p=*0.701 |
